# Experiences of children with central venous access devices: a mixed-methods study

**DOI:** 10.1038/s41390-022-02054-3

**Published:** 2022-04-11

**Authors:** Amanda J. Ullman, Tricia M. Kleidon, Victoria Gibson, Mari Takashima, Jessica Schults, Paula Cattanach, Rebecca Paterson, Marie Cooke, Joshua Byrnes, Masnoon Saiyed, Vineet Chopra, Claire Rickard

**Affiliations:** 1 School of Nursing, Midwifery and Social Work, University of Queensland, Brisbane, QLD Australia; 2 Children’s Health Queensland Hospital and Health Service, Brisbane, QLD Australia; 3 Menzies Health Institute Queensland and School of Nursing and Midwifery, Griffith University, Brisbane, QLD Australia; 4 Herston Infectious Disease Institute, Metro North Hospitals and Health Service, Brisbane, QLD Australia; 5 Centre for Applied Health Economics, Menzies Health Institute Queensland, Griffith University, Brisbane, QLD Australia; 6 Department of Medicine, University of Colorado at Denver, Anschutz Medical Campus, Denver, CO USA

## Abstract

**Background:**

Our study aims to explore the experience of having a central venous access device (CVAD) from the perspective of the child and family and how movements within and outside of hospital environments influence this experience.

**Methods:**

A mixed-methods study was conducted across Children’s Health Queensland (Australia), including inpatient and home-care settings. Children less than 18 years with CVADs were eligible and followed for 3 months or CVAD removal. A subgroup of primary caregivers participated in semi-structured interviews. Quantitative and qualitative measures of child and family CVAD experiences were explored.

**Results:**

In total, 163 patients with 200 CVADs were recruited and followed for 6993 catheter days (3329 [48%] inpatients; 3147 [45%] outpatients; 517 [7%] home). Seventeen participants were interviewed. Experiences of having a CVAD were complex but predominantly positive primarily related to personalized CVAD care, healthcare quality, and general wellbeing. Their experience was shaped by their movements through hospital and home environments, including care variation and distress with procedures. Device selection and insertion location further influenced experience, including safety, impairments in activities of daily living, school, and recreation.

**Conclusions:**

CVAD experiences were influenced by nonmodifiable (e.g., diagnosis) and modifiable factors (e.g., education; care variation). Clinical approaches and policies that account for family and child considerations should be explored.

**Impact:**

- Variation in decision making and management for pediatric CVADs is accepted by many clinicians, but the influence this variation has on the health experience of children and their families is less well explored.
- This is the first study to draw from a broad range of children requiring CVADs to determine their experience within and outside of healthcare facilities.
- Interdisciplinary clinicians and researchers need to work collaboratively with children and their families to provide resources and support services to ensure they have positive experiences with CVADs, no matter where they are managed, or who they are managed by.

## Introduction

There is no innocuous reason for a child to need a central venous access device (CVAD). These devices are often used for the treatment of the most complex and life-threatening conditions. Some indications are relatively short, for example, administration of inotropes and fluids during cardiac procedures. However, most indications are prolonged, for example, hemodialysis, chemotherapy, or parenteral nutrition.^1,2^ All reasons for CVAD insertion are life-changing for the child and their family or caregiver.^3^

The selection, insertion, and management of CVADs span traditional health disciplines.^4^ Children enter the health system via multiple departments (e.g., emergency, admissions), are managed by several sub-specialties (e.g., cancer care, cardiology, gastroenterology, trauma), and have CVAD insertion procedures carried out by further specialist groups (e.g., interventional radiologist, surgeons, anesthesiologists, intensivists). Additional support and advice are provided by further specialists (e.g., infectious disease, pharmacy), with management by multiple caregivers the norm (e.g., nurses from each of the specialty areas, parents, and carers). Variation in decision making and management for pediatric CVADs is accepted by many clinicians, but the influence this variation has on the health experience of children and their families is less well explored.

With the move from hospital- to home-based care, pediatric CVADs now cross even more borders. Children and their families are the primary manager of their devices in the home setting, especially for life-long vascular access dependent conditions, such as gut enteropathy requiring parenteral nutrition.^3^ Even for acute illnesses, outpatient parenteral antimicrobial therapy is the new standard of care for the treatment of many infections.^5^ However, having a complex and complication-prone device in the community setting may be challenging for many children and families. In the middle of these two extremes is the experience of children frequently moving in and out of hospital settings, for example, children receiving cancer treatment. Absent from the literature is an exploration of how these setting changes influence the experience of children with CVADs and their families, including the care they are provided. Therefore, we aimed to explore the experience of having a CVAD from the perspective of the child and family, and to explore how movements within and outside of hospital environments further influence this experience.

## Methods

### Study population and design

A mixed-methods, single-center study of children who had a CVAD inserted was undertaken between September 2018 and March 2020.^6^ Using a convergent design (Quan + Qual),^7^ quantitative data were collected bi-weekly, until either CVAD removal or up to three months. Concurrently, qualitative data were collected via semi-structured interviews of a subgroup of children and their parents. Ethics approval was obtained from the Children’s Health Queensland and Griffith University Human Research Ethics Committee (HREC/18/QRCH/19; 2018/096). The study is reported in accordance with the COREQ^8^ and STROBE^9^ reporting guidelines.

### Setting

The study was conducted across Children’s Health Queensland (Australia), including hospital (Queensland Children’s Hospital: QCH) and home-care settings. QCH is a 359-bed pediatric metropolitan hospital in Queensland that provides tertiary referral care to patients from birth to 18 years of age. All clinical care areas were included in this study, including the hospital in the home and outpatient services.

### Participants and sample size

All children aged up to 18 years and undergoing insertion of a CVAD at the hospital were eligible for inclusion. Patients requiring other vascular access types (e.g., peripheral intravenous catheter), and sub-specialty devices (e.g., extracorporeal membrane oxygenation cannulae) were excluded. Due to local resources and to ensure quality follow-up (minimizing missing data), a maximum of ten participants could be followed at a time. Whenever a patient finished follow-up (at study end [3 months or device removal]), a new patient was enrolled. As described in the accompanying paper,^10^ the sample size of the quantitative data was based on benchmarking of complications against international literature (200 CVADs; allowing 5% absolute precision, 90% confidence, to establish a predicted 25% failure).^11^

A subgroup of primary caregivers (e.g., parents, legal guardians) took part in the semi-structured interviews. The sample size for the subgroup was not defined a priori, and data were gathered using purposive sampling until thematic saturation was achieved.^12,13^ This was determined through field notes where the salient issues of each interview were noted and reviewed throughout the interview period by two reviewers. In a reflexivity exercise, field note summaries were presented to all parent interviewees providing a degree of trustworthiness and confirmability of findings.

### Measures of patient experience and covariates

At the time of study commencement, there were no reliable and valid patient experience/quality of life measures suitable to child and family CVAD experiences.^14^ Hence, we collected a range of quantitative and qualitative data to explore this phenomenon. Quantitative variables were prospectively collected on the entire cohort. This included data surrounding the patient (e.g., age, primary diagnosis), CVAD (e.g., type), movement (e.g., where the child’s CVAD is mainly being managed; between hospital inpatient, outpatient, or at home), and utility.^15–17^ The impact of having a CVAD on pain and discomfort was reported by children (if >10 years) or by parents (if < 10 years) via ten-point numeric rating scales [NRS] with standardized anchors.^18,19^ CVAD-related experiences in activities of daily living, patient-reported outcomes, and strategies for CVAD pain and anxiety were reported (by child/parent) via yes/no.^14,20^

Qualitative data, captured via interviews, were used to triangulate these data across the broader experience of children and family with CVADs.

### Data sources

Operating theater lists were screened daily by the Research Nurse (ReN) to assess eligible patients. The patients were assessed bi-weekly (twice a week) by the research nurses either in person (while still admitted to QCH) or over the phone (while discharged or at an alternative site),^21^ with additional data sourced from the patient’s electronic medical record. All experience measures were first collected by a discussion with the child, if >10 years of age and able to communicate; and if not, by parental report. All data were collected using a dedicated, secure, web-based REDCap (Research Electronic Data CAPture, Vanderbilt) database.^22^

Prior to interviews, legal guardians were approached face-to-face and asked to participate, and written informed consent was obtained. Interviews were recorded, anonymized, transcribed, and thematically analyzed.^23^ All interviews were conducted by authors VG and PC, who were the study ReNs and are pediatric nurses with specialized training in conducting interviews. The study ReNs were independent of participants’ treating teams, allowing for candid discussion, and the relationship between the ReNs and participants facilitated gaining a deeper understanding of participants’ experiences of having a CVAD while ensuring a professional relationship.^24^ To ensure consistency, a semi-structured interview guide was used to elicit participants’ views, including questions such as “tell me about your experience with your CVAD”. The interview guide was informed by previous research and evidence synthesis^25–29^ and informal discussions with clinicians and consumers. Interview questions broadly focused on participants’ personal experience with CVADs (Supplementary Material 1) and included open-ended questioning. Follow-up prompts were designed to lead participants to recount their personal experiences and could be adapted based on participant responses.^30^ Pilot testing with families was not undertaken but questions were tested on clinicians (*n* = 5) caring for families across sites.^30^ All interviews were audio recorded and independently transcribed verbatim for accuracy.^24^ Transcripts were not returned to participants for comment or correction. Interview duration was approximately 20 min.

### Bias

An equitable inclusion criterion was used to minimize selection bias. Information bias was decreased by having data collected by dedicated, experienced ReNs (including established inter-rater reliability of quantitative data collection processes),^21^ clear and rigorous definitions, and prospective methods (eliminating recall bias).^31^

### Data analysis

Quantitative data collected were cleaned and checked for accuracy (10% by second ReN) prior to importing into Stata (version 13; StataCorp, College Station, TX) for analysis. Patient, CVAD, location, management, and impact characteristics are descriptively reported, using categorical and continuous descriptors appropriate to their distribution. Catheter days were used as a denominator since this study aims to demonstrate the patient journey between the settings and each patient could have been in multiple settings with the same device. We assumed that each observation continued until the next assessment. The alluvial diagram to visualize CVAD setting pathways was developed using RAWGraphs. All other figures were made using Tableau 2021.1.

Qualitative interview data were analyzed using inductive thematic analysis.^32^ Analysis was undertaken as per Braun and Clarke’s six phases of thematic analysis.^32^ Initially, two researchers (J.S. and V.G.) read transcribed interviews and independently generated initial codes. Line-by-line coding was used (facilitating an audit trail) to enhance dependability.^33^ Codes were then used to inform concept formation, and themes and subthemes were identified by consensus between researchers. Themes were reviewed and defined with continued reference to codes and raw data via discussion with the project team to enhance authenticity.^12^ A number of strategies were used to enhance data quality and increase rigor, including data immersion and triangulation of emerging findings between researchers.^34^

Standard phases of thematic analysis (i.e., familiarization, deductive code generation, searching, reviewing, and defining the identified themes) were employed.^35,36^ Themes and subthemes were identified by consensus (authors J.S., A.J.U., and V.G.). These data were then integrated and triangulated, to explore and compare the quantitative and qualitative results.^6,7^

## Results

### Participants: quantitative

**Fig. 1:**
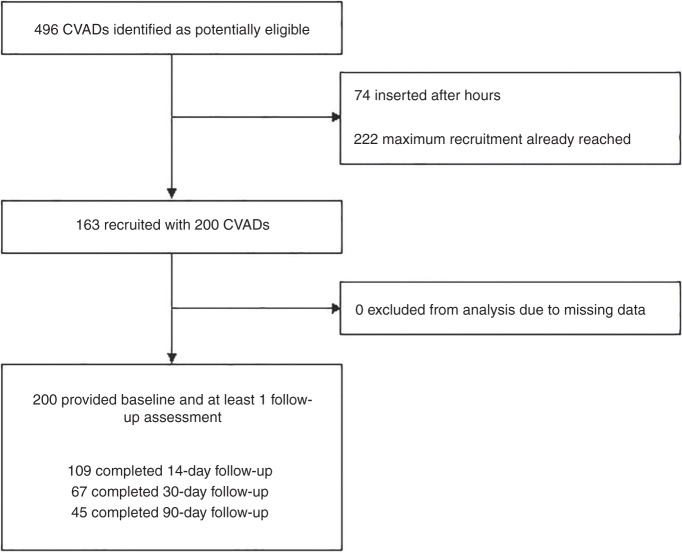
CONSORT Flow diagram of study participation. CVADs Central venous access devices.

As described in Fig. 1, 496 CVADs were inserted over the study period. From this sample, 296 could not be recruited due to the maximum follow-up sample (*n* = 10 per time) being reached. Finally, 163 patients with 200 CVADs were recruited and followed for 6993 catheter days (3329 [48%] hospital days; 3147 [45%] outpatient days; 517 [7%] in home settings).

**Fig. 2:**
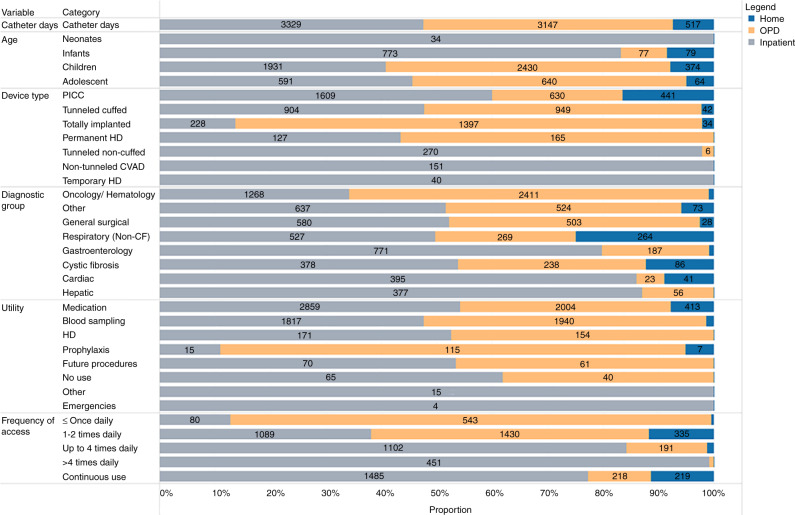
Participant characteristics, including variations in patient, device, and utility across settings (per catheter days). CF cystic fibrosis, CVAD central venous access device, HD hemodialysis, OPD outpatient departments, PICC peripherally inserted central catheter. Catheter days (per setting) were derived from bi-weekly observations, assuming that each observation continued until the next assessment. Six participants had devices removed prior to the first assessment.

Participants in the study included a balance of age, diagnostic, and catheter types, but movement between settings was based on multiple characteristics (Fig. 2 and Supplementary Table 1). Neonates (100%; 34 catheter days) and infants (83%; 773 catheter days) were primarily managed in hospitals, but children and adolescents were primarily managed in outpatient departments and at home. Most children (all ages) with oncological and hematological diagnoses were managed in outpatient departments (65%; 2411 catheter days). Home care was chiefly used for children with respiratory conditions (264 catheter days), and most children managed in outpatient and home care settings had PICCs, totally implanted venous devices, or tunneled cuffed CVADs.

The utility of the CVAD varied extensively across settings. For children in home care, CVADs were primarily used for the administration of medications (413 catheter days), especially antibiotics (388 catheter days). Children requiring very frequent access (>4 accesses per day) were principally (99% of catheter days) managed in hospital inpatient settings.

**Fig. 3:**
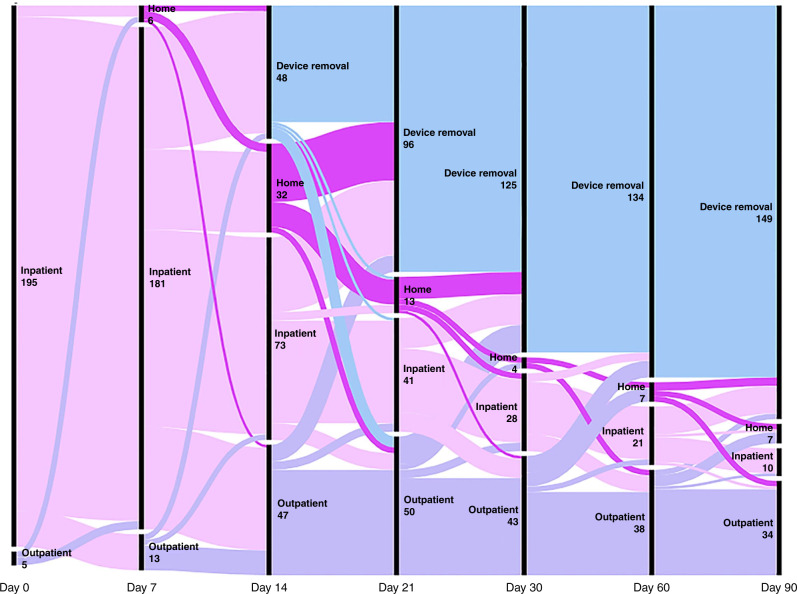
CVAD setting pathway, across time intervals (200 CVADs; 6993 catheter days). CVAD movement between settings is demonstrated by proportional flow patterns. Each line on the alluvial diagram represents a CVAD (*n* = 200), with *x*-axis = day of assessment, and *y*-axis = setting (i.e., inpatient, home, outpatient, device removal).

While children with CVADs moved extensively within and outside of hospital settings, they primarily started in inpatient hospital settings, with outpatient care relatively frequent after the first week. Movement between home- and hospital-based care by individuals was common (Fig. 3).

### Main results: quantitative

**Fig. 4:**
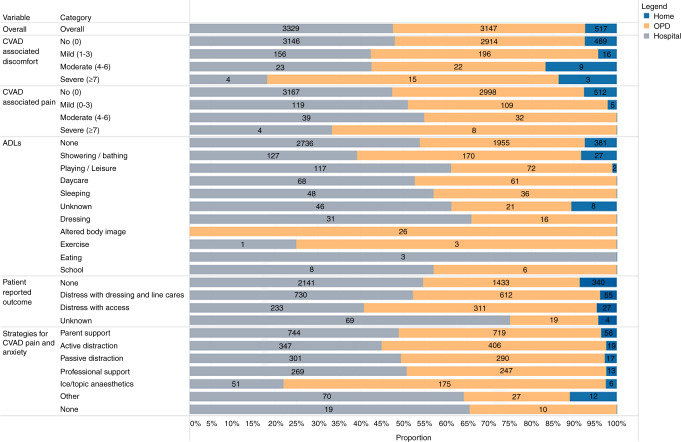
CVAD-associated experience, including comparisons across settings (per catheter days). CVAD central vascular access device, ADL activity of daily living. Catheter days duration of each setting was derived from bi-weekly observations and assuming that each observation continued until the next assessment.

Figure 4 displays the quantitative experience measures of the children, per CVAD and per CVAD day (Supplementary Table 2). In general, most children reported no (0/10 NRS; 93.7% and 95.5% of CVAD days) to mild (1–3; 5.3% and 3.3% of CVAD days) discomfort and pain associated with their CVAD, no matter their setting. However, there were consistent reports of difficulties with activities of daily living, specifically showering/ bathing (4.6% of CVAD days) and playing/leisure (2.7% of CVAD days). Most of the CVAD-related impacts on activities of daily living were evident in the children being managed in outpatient departments, including influencing their ability to play, sleep, dress, and attend school or day care. Distress with dressing and line care was consistently reported, across all settings (20% of CVAD days). The strategies to relieve CVAD-associated pain and anxiety were inconsistent, but parent support and distraction were most common (21.7% of CVAD days), in comparison to professional support (7.6% of CVAD days) or topical anesthetics (3.3% CVAD days).

### Participants: qualitative

We interviewed 17 individuals (2 mother-father dyads and 13 individual parents) from the 163 cohorts (Fig. 1). Children were primarily receiving treatment associated with a chronic disease diagnosis (*n* = 15), or requiring inpatient or outpatient-based hospitalization as often as tri-weekly (e.g., renal replacement therapy) to monthly (e.g., cancer therapy).

### Main results: qualitative

#### Perceptions and experiences of families

**Fig. 5:**
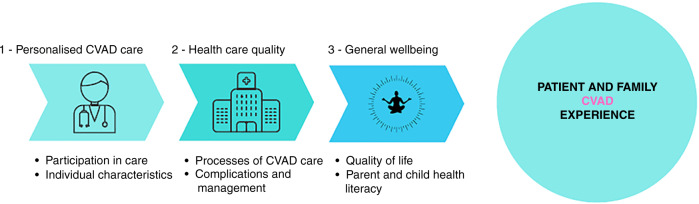
Thematic map of perceptions and experiences of children and families. CVADs Central venous access devices.

Most families held positive views about their child’s CVAD experience. Three major themes were identified: (1) personalized CVAD care; (2) healthcare quality; and (3) general wellbeing. Figure 5 depicts the final themes and subthemes.

### Personalized CVAD care

This theme incorporated two subthemes: participation in care and Individual characteristics.

Participation in care was highlighted as an important consideration, with parents valuing involvement in the planning for and care of their child’s CVAD. This included key decisions regarding device selection and aspects of service provision, with one mother stating “PICCs are much better for her, the port has never been (good for her), it was like her body rejected it (clots)” (P01). Participants viewed shared decision making as vital to improve clinical experience, outcomes, their overall device confidence, and general health experience. However, families also felt there was limited or absent discussion of their child’s long-term vascular access plan. Despite parents alluding to discretionary plans in the event of device failure “if the midline fails, he will likely need a port” (P01) parents discussed these plans could go in “different directions” (P07) and it is “their (treating team) choice at the end of the day” (P08), resulting in uncertainty regarding long-term treatment plans.

Parents also revealed that their child’s individual characteristics, including age “as she gets bigger, she will get stronger” (P05) and underlying condition (e.g., “congenital nephrotic syndrome” (P04)), were major contributory factors in their CVAD experience. Parents discussed a chronic diagnosis meant the type of CVAD required was not “up for discussion”, “he required a PICC for T-cell acute lymphoblastic leukemia… [they are] only good for a month or so” (P02). With children’s age and diagnoses differing across families, it was evident that parents with younger children or prolonged treatment regimens (e.g., “Ewing sarcoma, 9-10 months of expected treatment” (P06)) faced additional challenges with their CVAD and transitions through care.

### Healthcare quality

Two subthemes were contained within this overarching theme: processes of CVAD care and complications and management.

Processes of CVAD care including routine procedures such as taking bloods, antibiotic treatment, and dressing changes were consistently highlighted. The value of a CVAD in relation to blood sampling was frequently alluded to with one mother stating, “they were able to access the bloods without having to stab him, so that was good” (P09). However, other procedures, such as CVAD dressing changes, were frequently described as challenging “we’ve had some pretty bad ones” (P10) and “it’s the dressing change he’s most worried about” (P14). However, families reported valuing support services such as occupational therapy. Distraction strategies learnt as an inpatient were identified by some participants as valuable when transitioning and commencing CVAD cares at home “he’s remembered some of them and used them (at home)” (P02).

Clinical practice variation across healthcare settings was discussed by some families, who observed healthcare provider “confusion” (P08) regarding best practice when transitioning between clinical areas, hospitals, and primary care providers. One family expressed that they had “had a few dramas” (P10) with regional centers perceived to have reduced access to the clinical expertise, equipment, and “dressing” supplies needed for desired service provision. The importance of provider clinical competencies regarding pediatric CVAD care was also highlighted by families including one participant who stated,

“you feel like you need to be paying attention and watching to make sure that somebody doesn’t, that it’s always being looked after at the standard it needs … it was challenging for us too, because you don’t want to be that person“ (P13).

Most families expressed concern regarding the potential for device complications and failure, specifically “infection” (P01, P04, P05, P11), blood “clots” (P01, P06, P08, P10), “fracture and dislodgement” (P05) and “extravasation” (P07). One family viewed the occurrence of a CVAD-associated infection as an “unpreventable, inevitable” (P05) complication. The majority of parents identified substantial burden was associated with CVAD complications including treatment delays, insertion of new devices “going under, in a surgical environment..at night.. a bit scary” (P12) and financial consequences including “petrol” (P01) costs associated with coming in for anti-thrombosis treatment. Many parents expressed concern regarding the long-term impact of vessel health and CVAD failure, expressing concern over “the permanent damage it can do to his veins” (P10).

### General wellbeing

This theme encompassed: quality of life and parent and child health literacy.

Participants described the insertion of a CVAD as “necessary” to facilitate “life saving treatment” across hospital and home settings, with one parent commenting it meant their child could “stay alive” (P05). The impact of the CVAD on the child’s quality of life was discussed by most parents who believed, while “it just complicates little daily things” (P07) like “showering” (P15), overall parents believed their children were resilient and tolerated the CVAD well. Parents discussed CVADs impacting day-to-day life including “she has to have a million photos a day to make sure that it (CVC) still looks ok … it doesn’t affect her at school” (P08) and “going to the toilet…” (P08). Parental strength and child resilience were evident throughout the interviews with statements such as “sometimes it’s hard to do it, but they (children) know that mummy can do it” (P01) and “needles bruise, they hurt’ but he’s tough” (P01).

Parents consistently described the value of health literacy to improve their CVAD experience. They discussed the need for the healthcare facility to provide “a bit more education” (P11) or a package of information, for example, diagnostic and treatment counseling that fulfills the needs of each family, especially across rural and regional settings. Parents also highlighted the need for systems that support parents and where appropriate adolescents to become knowledgeable about their own CVAD and provide information on how to obtain further information and health service support. This was particularly relevant for the early days post CVAD insertion “if we had been in earlier in our journey, we may not have been so confident” (P13). Parents expressed gratitude for the support they had received as inpatient and peer support networks on social media platforms and connections established with other families they met during their hospitalization.

## Discussion

Despite the complexities associated with having a CVAD and navigating healthcare, children and parents within this study reported primarily positive experiences. Across hospitalization and home care, they pragmatically focused on the ‘need’ for the device and the key requirements to maintain its patency and function, a finding similar to what has been seen in previous adult studies.^37,38^ However, their experience can be negatively impacted by several factors, including inconsistent management, inadequate support, and potential or actual complications. These data have provided opportunities to practically improve the families experience of having a CVAD, especially whilst moving between care settings. This is particularly important for children with a chronic illness or complex medical needs who experience frequent healthcare interactions and movements between the hospital and the home, with a CVAD.

Many major negative influences on the experience of children and families centered around inadequate support, inconsistency in care provision, and fear of adverse events. These insights provide an opportunity to target practical strategies to tangibly improve future experiences. Some of these are more difficult issues to solve. For example, inconsistency in care provision is often based on a low evidence-base to inform clinical decision making. Also, the fear of adverse events associated with CVADs is a credible fear, considering the frequent high rates of CVAD complications in pediatrics, and the devastating sequelae.^26,29^ Research evaluating everyday management of CVADs to improve care provision and reduce adverse events across health systems is minimal, especially in pediatrics,^39^ and translation is slow. This will take time. However, an additional strategy to improve practice consistency and research implementation is to put children and their families in the center of the CVAD experience, by improving their knowledge, and facilitating shared decision making.^40^ This is a solution shared by the parents included in the study, requesting support in the form of focused educational materials, inpatient support and referral linkages, as well as outreach facilities for regional and rural families. This should be a priority focus for health systems to develop.

Procedure-associated pain, stress, and discomfort significantly negatively impact the health experiences of children with CVADs, and their families. Clinical procedures, such as dressing changes and line care, are necessary, but the prevention of pain, stress, and discomfort during the procedure requires a multi-tiered, multi-disciplinary approach, combining pharmacological and non-pharmacological agents. The “Children’s Comfort Promise” is an example of how this can be achieved, involving a system-level implementation of a standard of care for needle procedures.^41^ In addition, while there is low- to very-low-quality evidence to support the use of individual psychological interventions, including distraction, hypnosis, cognitive behavioral therapy, and breathing, to reduce pain and distress,^42^ much current research is focusing on the use of virtual reality as practical, non-pharmacological adjuvant analgesia.^43,44^ This is a component of practice at the center of considerable innovation, which needs to be rapidly implemented into care to improve the experiences of children with CVADs.

A key finding of the study was the commonality in the characteristics of children and devices that could be transitioned between inpatient care to home-based care. Home is not a safe place for all CVAD types; with some devices (e.g., non-tunneled CVADs) reserved only for inpatient use.^2^ Similarly, older children (>1 year) and those with long-term health conditions (e.g., cystic fibrosis) more frequently moved between inpatient, home, and outpatient services. This is likely to be reflective of many contributing factors, including diagnosis, device stability, child and parental health literacy and confidence, and clinical services decision making. It is likely that, with increased health infrastructure, technical resources, and support, further populations and devices could be used across care settings, potentially further improving clinical outcomes and health experiences.

Importantly, we found that device selection influences patient experience. Recent pediatric CVAD guidelines^2,3^ highlight the importance of child and parental participation during device selection and future planning. This was further demonstrated within this study and previous studies in adult cancer care,^38^ with parents stressing the influence of devices types and insertion location on everyday experiences, such as activities of daily living, school, and procedural management. Future studies could further examine the perspectives of patients and caregivers, to explore the nexus of underlying diagnoses, complications encountered, and CVAD failure on device selection priorities. Demonstrated in our accompanying paper, device selection also impacts clinical outcomes, with complication rates varying between device types.^29^ Clinicians and families need to work together to ensure the devices and insertion sites being selected, meet the needs of the child, the child’s treatment, and the family. For children with chronic and complex health conditions, this includes the ability to self-manage in the home and enjoy childhood experiences, without adverse events.^3^

### Limitations

Our study has limitations. The scope of the study enabled the ascertainment of child and family perspectives only in a single center in Australia; therefore, the generalizability of study findings to healthcare facilities internationally warrants further research. In addition, the interviews were conducted with a smaller cohort, primarily with families experiencing chronic health conditions, so the results may not be applicable outside of this cohort. However, the mixed-methods approach allows the data to provide validation for each other and help draw meaningful conclusions about the study question, strengthening reliability.^7^ Across the study, high-quality research practices were followed, including a priori research questions, prospective design, and reporting procedures.

## Conclusions

Children and families report primarily positive experiences with their CVAD across health and home care environments. However, CVAD-associated adverse events and inconsistencies in care provision remain inter-related and significant sources of stress and harm. Areas of research and innovation are evident, and each requires elements of co-development and collaboration between children, families, clinicians, and healthcare services. In particular, investments should be made to better integrate consumer participation in care, explore and improve the practical impacts of CVADs on daily life outside the hospital, and the provision of education and other supportive structures for families. A CVAD should provide a reliable mechanism for treatment administration, not an additional stressor for the child or family.

## Supplementary information

Supplementary material

Supplementary Table 2

## Data Availability

The data that support the findings of this study are available on reasonable request from the corresponding author. The data are not publicly available due to privacy and ethical restrictions and therefore permission will need to be sought by the ethics committee that approved this study.
