# Supplementary material for: Experiences of children with central venous access devices: a mixed-methods study
Source: Pediatr Res. 2022 Apr 11;93(1):160–7. doi: 10.1038/s41390-022-02054-3 (PMC9876783; doi:10.1038/s41390-022-02054-3)
Supplement: Supplementary file 1 — Supplementary material [file 41390_2022_2054_MOESM1_ESM.pdf]

Supplement Table 1. Participant characteristics, including variations in patient, device and utility across settings (per catheter days)

Variable	Category	Inpatient n (%; Row)	OPD n (%; Row)	Home n (%; Row)	Total
**Catheter days**		**3329 (47.6)**	**3147 (45.0)**	**517 (7.4)**	**6993**
**Patient characteristics**					
**Age**	Neonates	34 (100.0)	0 (0.0)	0 (0.0)	34
	Infants	773 (83.2)	77 (8.3)	79 (8.5)	929
	Children	1931 (40.8)	2430 (51.3)	374 (7.9)	4735
	Adolescent	591 (45.6)	640 (49.4)	64 (4.9)	1295
**Diagnostic group**	Oncology/ Hematology	1268 (34.2)	2411 (64.9)	34 (0.9)	3713
	Other	637 (51.6)	524 (42.5)	73 (5.9)	1234
	General Surgical	580 (52.2)	503 (45.3)	28 (2.5)	1111
	Respiratory (Non-CF)	527 (49.7)	269 (25.4)	264 (24.9)	1060
	Gastroenterology	771 (79.8)	187 (19.4)	8 (0.8)	966
	Cystic Fibrosis	378 (53.8)	238 (33.9)	86 (12.3)	702
	Cardiac	395 (86.1)	23 (5.0)	41 (8.9)	459
	Hepatic	377 (87.1)	56 (12.9)	0 (0.0)	433
**Device characteristics**					
**Device type**	PICC	1609 (60.0)	630 (23.5)	441 (16.5)	2680
	Tunneled cuffed	904 (47.7)	949 (50.1)	42 (2.2)	1895
	Totally implanted	228 (13.7)	1397 (84.2)	34 (2.0)	1659
	Permanent HD	127 (43.5)	165 (56.5)	0 (0.0)	292
	Tunneled non-cuffed	270 (97.8)	6 (2.2)	0 (0.0)	276
	Non-tunneled CVAD	151 (100.0)	0 (0.0)	0 (0.0)	151
	Temporary HD	40 (100.0)	0 (0.0)	0 (0.0)	40
**Utility**					
**Utility[a]**	Medication	2859 (54.2)	2004 (38.0)	413 (7.8)	5276
	Blood sampling	1817 (47.7)	1940 (50.9)	54 (1.4)	3811
	HD	171 (52.6)	154 (47.4)	0 (0.0)	325
	Prophylaxis	15 (10.9)	115 (83.9)	7 (5.1)	137
	Future Procedures	70 (53.4)	61 (46.6)	0 (0.0)	131
	No use	65 (61.9)	40 (38.1)	0 (0.0)	105
	Other	15 (100.0)	0 (0.0)	0 (0.0)	15
	Emergencies	4 (100.0)	0 (0.0)	0 (0.0)	4
**Frequency of access**	≤ once daily	80 (12.8)	543 (86.7)	3 (0.5)	626
	1-2 times daily	1089 (38.2)	1430 (50.1)	335 (11.7)	2854
	up to 4 times daily	1102 (84.2)	191 (14.6)	16 (1.2)	1309
	>4 times daily	451 (99.1)	4 (0.9)	0 (0.0)	455
	Continuous use	1485 (77.3)	218 (11.3)	219 (11.4)	1922

CF: Cystic fibrosis; CVAD: Central venous access device; HD: Hemodialysis; OPD: Outpatient departments; PICC: Peripherally inserted central catheter
Catheter days (per setting) were derived from bi-weekly observations, assuming that each observation continued until next assessment. [a]6 participants had devices removed prior to first assessment

Supplement Table 2. CVAD-associated experience, including comparisons across settings (per catheter days)

		Inpatient			OPD			Home			Total	
**Variable**	**Category**	**Days**	**Days % (Row)**	**Days % (Col)**	**Days**	**Days % (Row)**	**Days % (Col)**	**Days**	**Days % (Row)**	**Days % (Col)**	**Overall Days**	**% Col**
**Overall**		3329	47.6	100.0	3147	45.0	100.0	517	7.4	100.0	6993	100.0
**CVAD associated discomfort**	No (0)	3146	48.0	94.5	2914	44.5	92.6	489	7.5	94.6	6549	93.7
	Mild (1-3)	156	42.4	4.7	196	53.3	6.2	16	4.3	3.1	368	5.3
	Moderate (4-6)	23	42.6	0.7	22	40.7	0.7	9	16.7	1.7	54	0.8
	Severe (≥7)	4	18.2	0.1	15	68.2	0.5	3	13.6	0.6	22	0.3
**CVAD associated pain**	No (0)	3167	47.4	95.1	2998	44.9	95.3	512	7.7	99.0	6677	95.5
	Mild (0-3)	119	51.1	3.6	109	46.8	3.5	5	2.1	1.0	233	3.3
	Moderate (4-6)	39	54.9	1.2	32	45.1	1.0	0	0.0	0.0	71	1.0
	Severe (≥7)	4	33.3	0.1	8	66.7	0.3	0	0.0	0.0	12	0.2
**ADLs**	None	2736	53.9	82.2	1955	38.5	62.1	381	7.5	73.7	5072	72.5
	Showering / bathing	127	39.2	3.8	170	52.5	5.4	27	8.3	5.2	324	4.6
	Playing / Leisure	117	61.3	3.5	72	37.7	2.3	2	1.0	0.4	191	2.7
	Daycare	68	52.7	2.0	61	47.3	1.9	0	0.0	0.0	129	1.8
	Sleeping	48	57.1	1.4	36	42.9	1.1	0	0.0	0.0	84	1.2
	Unknown	46	61.3	1.4	21	28.0	0.7	8	10.7	1.5	75	1.1
	Dressing	31	66.0	0.9	16	34.0	0.5	0	0.0	0.0	47	0.7
	Altered body image	0	0.0	0.0	26	100.0	0.8	0	0.0	0.0	26	0.4
	School	8	57.1	0.2	6	42.9	0.2	0	0.0	0.0	14	0.2
	Exercise	1	25.0	0.0	3	75.0	0.1	0	0.0	0.0	4	0.1
	Eating	3	100.0	0.1	0	0.0	0.0	0	0.0	0.0	3	0.0
	Moving	0	0.0	0.0	0	0.0	0.0	0	0.0	0.0	0	0.0
	Socialization	0	0.0	0.0	0	0.0	0.0	0	0.0	0.0	0	0.0
	None	2141	54.7	64.3	1433	36.6	45.5	340	8.7	65.8	3914	56.0

**Patient reported outcome**	Distress with dressing and line cares	730	52.3	21.9	612	43.8	19.4	55	3.9	10.6	1397	20.0
	Distress with access	233	40.8	7.0	311	54.5	9.9	27	4.7	5.2	571	8.2
	Unknown	69	75.0	2.1	19	20.7	0.6	4	4.3	0.8	92	1.3
**Strategies for CVAD pain and anxiety**	Parent support	744	49.0	22.3	719	47.3	22.8	56	3.7	10.8	1519	21.7
	Active distraction	347	44.9	10.4	406	52.6	12.9	19	2.5	3.7	772	11.0
	Passive distraction	301	49.5	9.0	290	47.7	9.2	17	2.8	3.3	608	8.7
	Professional support	269	50.9	8.1	247	46.7	7.8	13	2.5	2.5	529	7.6
	Ice/topic anaesthetics	51	22.0	1.5	175	75.4	5.6	6	2.6	1.2	232	3.3
	Other	70	64.2	2.1	27	24.8	0.9	12	11.0	2.3	109	1.6
	None	19	65.5	0.6	10	34.5	0.3	0	0.0	0.0	29	0.4

CVAD: Central vascular access device, ADL: Activity of Daily Living
